# COVID-19 measures in Belgium: how perception and adherence of the general population differ between time periods

**DOI:** 10.1186/s12889-022-12654-7

**Published:** 2022-02-07

**Authors:** Joris Adriaan Frank van Loenhout, Kirsten Vanderplanken, Stephan Van den Broucke, Isabelle Aujoulat

**Affiliations:** 1 Centre for Research on the Epidemiology of Disasters (CRED), Institute of Health and Society, Université catholique de Louvain, Clos Chapelle-Aux-Champs 30 B1.30.15, 1200 Brussels, Belgium; 2 Psychological Sciences Research Institute, Université catholique de Louvain, Place du Cardinal Mercier 10 bte L3.05.01, 1348 Louvain-la-Neuve, Belgium; 3 Centre for Health Promotion Knowledge Transfer (RESO), Institute of Health and Society, Université catholique de Louvain, Clos Chapelle-Aux-Champs 30 B1.30.15, 1200 Brussels, Belgium

**Keywords:** COVID-19, Risk perception, Adherence, Infection prevention and control measures, Risk factors, Belgium, Population survey, Questionnaire

## Abstract

**Background:**

Since the onset of the COVID-19 pandemic, Belgium has been hit by a series of surges in the number of COVID-19 cases. Each of these resulted in more stringent measures being taken to curb the pandemic. This study compared perception of and adherence to COVID-19 measures of the Belgian population at two time periods: September 2020 (survey 1) and April/May 2021 (survey 2).

**Methods:**

Two samples of approximately 2000 participants, representative for the Belgian population in terms of gender, age, province and socio-economic status, participated in an online survey. The survey questionnaire measured the perceived infection risk and severity, and the perception of and adherence to protective measures. Answers were compared between the time periods and risk factors for lower adherence were identified using multivariate linear regression.

**Results:**

In survey 2, at which time the measures were more stringent, respondents assessed the risk of infection for themselves as lower, and for parents and grandparents as higher than in survey 1. Scores for understanding and usefulness of the measures were higher in survey 2 compared to survey 1, while reported past and future adherence were lower. Risk factors for a lower adherence were being male, being young, speaking French vs. Dutch, and having undergone a symptomatic infection.

**Conclusions:**

It is important to consider the potential effect of fatigue among the population with regards to measures that are sustained for a long time, especially regarding measures related to social contacts. The identified risk factors for lower adherence offer insights to policy makers for future crisis communication regarding COVID-19.

## Background

The first case of COVID-19 was documented in December 2019, and up until the 3rd of August 2021, almost 200 million cases have been registered globally [1]. From the outset, an important strategy applied by most countries to limit the number of new COVID-19 infections was to implement infection prevention and control measures aimed at reducing transmission by means of e.g. social distancing [2]. In order to assess the effectiveness of such measures, many studies across the world have investigated the adherence of the general population or of specific groups. Studies from Africa [3–6], North America [7], Latin America [8], East Asia [9, 10], South Asia [11], the Middle East [12], Oceania [13] and Europe [14, 15] found varying rates of adherence. Factors that were found to be associated with lower adherence to COVID-19 measures include being male, belonging to a lower [7, 8, 16, 17] or a higher age group [18], being single [17], and coming from a disadvantaged background [8, 19], although the effects of these risk factors also depend on country-specific contexts.

The number of published studies that focused on the evolution of adherence to COVID-19 measures over time is much smaller. A German study showed that the proportion of respondents following safety behaviour during a second (lighter) wave was lower than during the first wave, and the second wave was also associated with higher levels of depressive symptoms [20]. A British study found a significant decline of the level of compliance with COVID-19 measures over a 5-month period, in accordance with a decreased stringency of the measures imposed by the government [21]. A longitudinal study among older adults in Switzerland found that future adherence is largely predicted by past adherence [22]. An Australian study reported generally higher levels of adherence at the start of a second wave, compared to the end of their first wave [13]. While these comparative or longitudinal studies provide important insights into the changes in adherence over time, it is also worthwhile to consider the factors that explain these changes in adherence. Apart from situational factors such as the severity of the outbreak or the duration of the measures, motivation is also likely to play a role. This is illustrated by a large comparative study in 14 countries, revealing an increase over time in adherence to ‘low-cost’ measures (such as mask wearing), but a decrease in adherence to ‘high-cost’ measures (such as social distancing). The latter was considered a sign of potential ‘pandemic fatigue’ [23].

Like many other countries, Belgium has been severely hit by the COVID-19 pandemic, with over 1.2 million registered cases until the 20th of September 2021 [24]. After the first case was detected on the 3rd of February 2020, the country has experienced several waves, each one associated with a reinforcement of the measures. The initial, most restrictive lockdown started in March 2020, and lasted until June 2020. This was followed by a summer that was relatively light in terms of severity of measures, with the population being allowed to see other people almost without restrictions. From September 2020 onwards, the number of cases started to rise again, which was responded to with increasingly stringent measures and finally resulted in a second lockdown from October 2020 until June 2021. During this period, citizens were allowed only one close contact in their house, while restaurants, cinemas and most sport facilities were closed. The measures that were applicable at a certain moment were continuously displayed on a government-controlled website, available in the official Belgian languages Dutch, French and German, as well as in English [25], and updates of the measures were communicated through periodic press conferences. Although we are not aware of any published studies comparing the adherence to COVID-19 measures in Belgium at different moments in time, reports of the periodic surveys undertaken by the Belgian National Institute of Health (Sciensano) showed that the proportion of the general population following the basic protective measures (e.g. restricting social contact, hygiene measures) was comparable between September 2020 and May 2021, while the proportion was lower in December 2020 [26]. A report based on surveys from University of Ghent and UCLouvain showed that the overall motivation to adhere was higher in September 2020 compared to April and May 2021 [27]. A follow-up survey from several Belgian universities showed that the proportions of respondents who had close contact with someone outside of their household were higher in April and May 2021, compared to September 2020 [28]. It should be noted that the aforementioned surveys relied on self-selection of participants, and did not include representative samples of the entire Belgian population.

For the current study, we had three main objectives. First, we compared the overall perceived risk of becoming infected and the perceived severity of an infection with COVID-19 between two time periods: September 2020, when the measures were relatively mild after a summer with relative freedom; and April/May 2021, during which time the measures were relatively restrictive and had persisted for a long time. Secondly, we compared the perception of, support for and level of adherence to the COVID-19 measures of citizens of Belgium for the two survey periods, with a view to identify potential contextual factors associated with levels of adherence. Thirdly, we assessed which personal characteristics were associated with lower levels of adherence. This type of information is important to improve targeted risk communication to specific groups.

## Methods

### Data collection

Our study design consisted of two cross-sectional surveys, undertaken between the 7th and 24th of September 2020 (first survey), and between the 28th of April and the 10th of May 2021 (second survey). Data were collected through internet surveys, by a specialised market research and opinion poll company. In each survey, we aimed for a sample size of ±2000. Respondents were selected so that the final samples were representative for adult citizens of Belgium in terms of gender, age, province and socio-economic status (composed of education and work status).

### Questionnaire

The study questionnaire was available in Dutch and French, and consisted of five sections: 1) demographic and socio-economic characteristics; 2) health status; 3) previous infection with COVID-19 and perceived or experienced consequences of contracting the disease; 4) use of information sources about COVID-19; 5) perception and implementation of protective behaviour related to measures currently in place. The latter section measured respondent’s reported understanding, perceived usefulness, ease to adhere, past adherence and (intended) future adherence of selected COVID-19 measures, rated on 5-point Likert scales (1 = ‘not at all understood/useful/easy/adhered to’; 5= ‘very well understood/useful/easy, or completely adhered to’). Overall, the questions were largely identical between both questionnaires, although questions that related to measures in place at the time of the survey were adapted accordingly. The questionnaire used in the first survey focused on eight measures, and the questionnaire in the second survey on ten. These measures related to different aspects of daily life that many people are confronted with (e.g. social contact, public and private events, work, shopping, mask wearing, travelling). Furthermore, we included questions assessing respondents’ overall support for the measures, including the COVID-19 vaccination strategy (in the second survey), rated on 5-point Likert scales (1 = strongly disagree; 5 = strongly agree). The full questionnaire of the first survey can be found in a previously published article [14]. Table 1 provides an overview of the measures we surveyed at each time period. The same measures on travel were still in effect in the second period. However, because the measures relating to social life were much more elaborate and stricter during that time, we decided to focus on these and not survey the travel measures to reduce the amount of questions asked. The category ‘symptoms’ was only surveyed in the second survey.

**Table 1:** Overview of measures effective in September 2020 and in April/May 2021, which were included in the surveys

Category	September 2020	April/May 2021
Social life	Social bubble limited to 5 persons	Having one close contact
	Private events limited to 10 persons	Receiving one close contact per household at the same time
	Official events limited to 200 persons indoors or 400 outdoors	Religious ceremonies with max. 15 persons
		Marriages with max. 15 persons
		Funerals with max. 50 persons
		Gathering outdoors in small groups
Work	Home working strongly recommended	Home working mandatory
Shopping	Shopping with max. 1 other person	Shopping with max. 1 other person
Public spaces	Face mask mandatory in public spaces	Face mask mandatory in public spaces
Symptoms		Testing and quarantine when symptoms
Travel	Travel form	
	Travel zones	

### Data analysis

All analyses were performed using IBM SPSS Statistics 27. Overall scores were calculated for understanding the preventive measures, perceived usefulness, ease to adhere, past adherence and future adherence, by averaging the individual scores for each measure. Outcomes and baseline characteristics for the two surveys were compared using Chi Square tests for categorical variables (gender, province, household composition, language, educational level, occupation, net annual household income, previous COVID-19 infection), and independent samples t-tests for continuous variables and those measured with Likert scales (age, expected and perceived health consequences, perceived risk of becoming infected, understanding, usefulness, ease to adhere, past and future adherence, support for measures). Correlations between past and future adherence for the different measures were determined by calculating Pearson’s r. Personal characteristics associated with past and future adherence were identified by undertaking multivariate linear regression, including the data from both survey periods. All potential predictors were included in the initial multivariate models, and factors that were not statistically significant (*p* > .05) were removed via a backward analysis. Survey period was kept as a confounder in the model, independent of its *p*-value.

## Results

### Descriptive statistics

The number of respondents in the first and second survey was 2008 and 1983, respectively. A comparison between the surveys in terms of demographic (gender, age, province, household composition) and socio-economic (educational level, occupation, net annual household income) characteristics gave no significant differences (Appendix 1 and 2). Also, native language skills did not vary between the two surveys (Appendix 3).

### COVID-19 and health consequences

There was a significant difference between the two surveys (*p* < .001) in the proportion of respondents that had had COVID-19, with the proportion of respondents that had not tested positive, nor had had symptoms indicative for a COVID-19 infection, being 85.1% in the first survey and 76.0% in the second (Table 2). The proportional increase was particularly high for respondents that had tested positive for COVID-19 and had shown symptoms but had not been hospitalised (1.3% vs. 6.5%). Similarly, the proportion of respondents who knew someone with a previous COVID-19 infection was higher in the second survey than in the first (Table 2). While COVID-19 vaccines were not available during the first survey period, 30.8% of respondents had received one dose of a vaccine when they participated in the second survey, and 7.1% were fully vaccinated.

**Table 2:** The number and proportion of respondents who previously had a COVID-19 infection in each survey round, and who know someone close to them who had a COVID-19 infection

Tested positive for COVID-19?	First survey		Second survey		Difference
	N	%	N	%	***p***-value^**1**^
					< .001
Not tested positive and no COVID-19 symptoms	1709	85.1	1508	76.0	
Not tested positive but had COVID-19 symptoms	198	9.9	209	10.5	
Tested positive but without COVID-19 symptoms	41	2.0	107	5.4	
Tested positive for COVID-19 symptoms but no hospitalisation	26	1.3	128	6.5	
Tested positive for COVID-19 symptoms and hospitalised	1	0.0	8	0.4	
Don’t know if tested positive for COVID-19	33	1.6	23	1.2	
**Someone close to you test positive for COVID-19?**	**N**	**%**	**N**	**%**	
Don’t know someone close with COVID-19	1391	69.3	884	44.6	
Know someone with COVID-19 symptoms but no positive test	147	7.3	110	5.5	
Know someone with positive COVID-19 test, not ill	87	4.3	236	11.9	
Know someone with positive COVID-19 test, ill but not hospitalised	242	12.1	732	36.9	
Know someone with positive COVID-19 test, hospitalised	118	5.9	203	10.2	
Don’t know if know someone with COVID-19	83	4.1	0	0.0	

^1^The p-value was obtained by undertaking a Chi Square test, as each respondent was classified in only one answer category. As the answer categories for the second question (did someone close to you test positive for COVID-19) were not mutually exclusive, a Chi Square test could not be undertaken.

Respondents who had not been ill and had not tested positive for COVID-19 (as well as those that answered ‘I do not know’) were asked to rate the expected health consequences on a scale of 0–100 (0 = ‘not at all severe’, 100 = ‘very severe’) should they become infected. This was the case for 1742 individuals in the first survey, and 1531 in the second. Their average scores were 57.3 (sd 27.5) and 59.6 (sd 27.5), respectively, which differed significantly (*p* = .017). The 266 respondents in the first survey and 452 in the second survey who (possibly) had been infected with COVID-19, which included individuals who tested positive but without any symptoms as well as persons who had COVID-19-like symptoms without a positive test results, were asked to rate how serious the consequences were that they had experienced, also on a scale of 0–100. The average scores for these groups were 35.5 (sd 29.4) and 33.2 (sd 28.8), respectively, which is not significantly different (*p* = .305). Finally, the health consequences were also assessed for those respondents who had had a confirmed COVID-19 infection with symptoms (*n* = 27 and 136 for the two surveys, respectively). The average scores for experienced severity of consequences for these groups were 51.4 (sd 26.0) and 45.8 (sd 26.5) for the first and second surveys, respectively, which again did not differ significantly (*p* = .319).

### Perceived risk of infection

When asked to indicate the risk of becoming infected with COVID-19 for themselves or for six other categories of people close to them on a 5-point Likert Scale ranging from ‘no risk’ [1] to ‘definite risk’ [5], respondents in the second survey considered their own vulnerability as significantly lower, and the vulnerability of older family members (parents and grandparents) as higher, when compared to respondents in the first survey. Other scores did not differ (Table 3).

**Table 3:** Experienced risk of becoming infected with COVID-19

	First survey		Second survey		Difference
Person(s)	N	Mean (sd)	N	Mean (sd)	***p***-value
Yourself	1986	2.85 (0.93)	1971	2.78 (1.00)	.011
Your parents	1347	2.84 (0.97)	1286	2.96 (1.07)	.004
Your grandparents	735	2.60 (1.09)	701	2.98 (1.26)	< .001
Your partner	1467	2.90 (0.94)	1452	2.83 (1.01)	.056
Your child (ren)	1345	2.94 (0.94)	1359	2.88 (0.96)	.130
A friend	1805	3.14 (0.84)	1767	3.14 (0.89)	.767
A close colleague	1286	3.16 (0.90)	1248	3.14 (0.92)	.644

### Perception of and adherence to measures

For each of the measures to protect against COVID-19 included in the questionnaire, respondents were asked to rate their understanding, perceived usefulness, ease to adhere, past adherence and (intended) future adherence on 5-point Likert scales. The results on questions related to measures during the first survey period have been presented elsewhere [14].

Scores for understanding: in the second survey, the measure of restricting the number of people present during a marriage was the least well understood, followed by the measures on receiving only one close contact at home and restricting the number of people present during religious ceremonies (Table 4). Measures that were best understood were the instruction to wear a face mask and to test and quarantine when presenting with COVID-19 symptoms. The two measures that were present in both questionnaires (wearing a face mask in public spaces, and shopping with maximum 1 other person) received higher scores for understanding in the second than in the first survey (4.74 vs. 4.50 and 4.44 vs. 4.38, respectively).

**Table 4:** Average scores in understanding, usefulness, ease to comply, past adherence and intended adherence for each of the 10 measures that applied during April / May 2021 (second survey period). The results on questions related to measures during the first survey period have been presented elsewhere [14]

Second survey	Understanding^**a**^	Usefulness^**a**^	Easy to adhere^**a**^	Past Adherence^**a**^	Intended adherence^**a**^
**Measure**	**Mean (sd)**	**Mean (sd)**	**Mean (sd)**	**Mean (sd)**	**Mean (sd)**
Having one close contact	4.11 (1.20)	3.70 (1.32)	2.95 (1.47)	3.86 (1.31)	3.76 (1.38)
Receiving one close contact per household	4.01 (1.25)	3.59 (1.36)	2.93 (1.46)	3.83 (1.33)	3.76 (1.38)
Religious ceremonies with max. 15 persons	4.08 (1.23)	3.64 (1.38)	3.73 (1.23)	4.30 (1.06)	4.34 (1.05)
Marriages with max. 15 persons	3.97 (1.26)	3.73 (1.33)	3.20 (1.44)	4.24 (1.12)	4.32 (1.06)
Funerals with max. 50 persons	4.10 (1.21)	3.70 (1.33)	3.39 (1.35)	4.34 (1.03)	4.40 (1.01)
Home working mandatory	4.09 (1.25)	4.24 (1.11)	3.66 (1.32)	4.06 (1.30)	4.10 (1.27)
Shopping with max. 1 other person	4.44 (0.99)	3.74 (1.33)	4.03 (1.12)	4.52 (0.90)	4.45 (0.95)
Face mask mandatory in public spaces	4.74 (0.69)	4.38 (1.10)	4.19 (1.17)	4.69 (0.71)	4.64 (0.79)
Gathering outdoors in small groups	4.33 (1.02)	3.99 (1.21)	3.91 (1.19)	4.33 (1.03)	4.28 (1.09)
Testing and quarantine when symptoms	4.55 (0.86)	4.59 (0.87)	4.07 (1.15)	4.55 (0.86)	4.60 (0.82)

^a^Higher scores indicate higher self-reported levels of understanding, perceived usefulness, etc.

Scores for perceived usefulness: overall, the levels for perceived usefulness of the measures were lower than for understanding in the second survey. The measures that were considered the least useful were the restriction to one close contact per household, restricting the number of people attending religious ceremonies to a maximum of 15, and restricting the number of people who could attend funerals to a maximum of 50 persons, and having one close contact. The highest scores for perceived usefulness were for testing and quarantine when presenting with symptoms, and wearing a face mask. When comparing the recurring measures for both surveys in terms of usefulness, the scores for wearing a face mask in public spaces were higher in the second survey (4.38) than in the first (4.16), as were the scores for shopping with maximum 1 other person (3.53 vs. 3.74 in first and second survey, respectively).

Scores for ease of adherence: on average, respondents gave lower scores for ease of adherence than for usefulness. The lowest score for ease of adherence was noticed for receiving one close contact at home, and the highest for wearing a face mask. The average ease of adherence score for wearing a face mask was higher in the second survey (4.19) than in the first (3.94), while the scores for shopping with maximum 1 other person were similar (4.00 and 4.03 in the first and second survey, respectively).

Scores for past and future adherence: apart from limiting close contacts to one and being allowed only one contact at home, all measures received scores that were higher than 4.00 for past and future adherence. The highest scores were again noted for wearing a face mask and for testing and quarantining when presenting with symptoms. The scores for past and future adherence were highly correlated for all measures, with Pearson r’s varying from .719 (funerals with maximum 50 persons) to .843 (having one close contact) (data not shown). For the recurrent measures, the average scores for past adherence were very similar between the surveys (4.68 vs. 4.69 for wearing a face mask in public spaces, and 4.55 vs. 4.52 for shopping with maximum one other person, respectively). For (intended) future adherence, the score for wearing a face mask in public spaces was similar between the surveys (4.61 in first vs. 4.64 in second), but the score for shopping with maximum one other person was slightly lower in the second survey (4.45) than in the first (4.55).

### Comparison of overall scores between the surveys

In order to compare the combined scores for the two survey rounds (Table 5), perceived and expected severity of infection were combined and transformed into the same scale as the other scores (minimum 1, maximum 5). There were no significant differences between perceived risk of becoming infected, perceived severity, and ease to adhere. For understanding the measures and perceived usefulness, the average scores were higher upon the second survey than upon the first. Yet, for past and (intended) future adherence, the scores were lower upon the second survey.

**Table 5:** Comparison of all outcome measures between the two surveys

Outcome measures	First survey^**a**^	Second survey^**a**^	Difference
	Mean (sd)	Mean (sd)	***p***-value
Perceived severity of infection	3.18 (1.15)	3.14 (1.20)	.371
Perceived risk of becoming infected	2.94 (0.75)	2.95 (0.78)	.740
Understanding	4.26 (0.81)	4.31 (0.78)	.042
Usefulness	3.83 (0.98)	3.94 (0.94)	< .001
Ease to adhere	3.71 (0.89)	3.66 (0.90)	.057
Past adherence	4.45 (0.73)	4.30 (0.76)	< .001
Intended adherence	4.39 (0.81)	4.26 (0.81)	< .001

^a^Higher scores indicate higher self-reported levels of the outcome measures.

### Support for measures and vaccination

When asked to rate the extent to which they agreed with statements on COVID-19 measures in general, on 5-point Likert scales (Table 6), respondents gave a significantly lower agreement score for the statement ‘the government should recommend, but not oblige adherence to the COVID-19 measures’ in the second survey than in the first. By contrast, the score for the statement ‘the government should oblige adherence to the COVID-19 measures’ was slightly higher in the second survey, although the difference was not statistically significant. A statement that was only included in the second survey, namely ‘the government should control adherence to the COVID-19 measures’ received higher scores than both statements mentioned previously. The average score for the statement ‘it is useful if the environment reminds me of COVID-19 measures (e.g. stickers on the floor)’ was significantly less agreed to in the second survey than in the first.

**Table 6:** Average scores in the first and second surveys on statements related to support for the COVID-19 measures and vaccines

Statement	First survey^**a**^	Second survey^**a**^	Difference
	Mean (sd)	Mean (sd)	***p***-value
Government should recommend measures	2.81 (1.39)	2.51 (1.42)	< .001
Government should oblige measures	3.94 (1.22)	4.01 (1.25)	.107
Government should control measures	–	4.08 (1.15)	–
Environmental reminders are helpful	3.85 (1.11)	3.77 (1.13)	.030
Important that everyone is vaccinated against COVID-19	–	4.17 (1.22)	–
COVID-19 vaccines protect yourself and others	–	4.26 (1.19)	–
COVID-19 vaccines are safe	–	3.58 (1.28)	–
COVID-19 vaccines prevent infection	–	3.82 (1.19)	–
COVID-19 vaccines prevent severe disease	–	4.02 (1.12)	–

^a^Higher scores indicate higher self-reported levels of the outcome measures.

Since vaccines to protect against COVID-19 were not yet available in September 2020, statements with regard to vaccination were only included in the second survey. The statements that received the highest scores were ‘I think COVID-19 vaccines are important to protect myself and others’ and ‘I find it important that everyone is vaccinated against COVID-19’. The lowest agreement scores were noted for ‘COVID-19 vaccines are safe’ and ‘COVID-19 vaccines are effective in preventing an infection’.

### Characteristics associated with adherence

There was a large similarity in personal characteristics associated with past and (intended) future adherence (Table 7). Men scored significantly lower than women. The youngest age group (18–30-year olds) scored significantly lower than all other age groups. In terms of occupation, only people who were incapacitated to work scored significantly different from the reference group of workers. Respondents who previously had had a confirmed, symptomatic COVID-19 infection scored significantly lower in terms of adherence than those who had not had a symptomatic infection. Language only contributed significantly to past adherence, in the sense that French-speaking respondents had lower adherence scores than Dutch-speakers. Lastly, the region where the respondent lived significantly contributed to (intended) future adherence, with scores for Wallonia being significantly lower than for Flanders and Brussels. Other characteristics that were tested as potential influencing factors, but which did not have a significant association, were annual income, health status at the moment of completing the survey, household composition and education level.

**Table 7:** Personal characteristics associated with past and future adherence to COVID-19 measures

Characteristic^**a**^	Past adherence^**b**^		Intended adherence^**b**^	
	B-value (CI)	p-value	B-value (CI)	***p***-value
Intercept	3.9 (3.8;4.1)	< .001	3.6 (3.5;3.8)	< .001
Language				
French	−0.2 (−0.2;−0.1)	< .001		
Dutch	Ref	Ref		
Region				< .001
Flanders			0.2 (0.1;0.2)	< .001
Brussels			0.1 (0.0;0.2)	.003
Wallonia			Ref	Ref
Gender				
Male	−0.1 (−0.1;−0.1)	< .001	-0.1 (− 0.1;0.0)	.008
Female	Ref	Ref	Ref	Ref
Age		< .001		< .001
18–30 years	Ref	Ref	Ref	Ref
31–45 years	0.2 (0.1;0.3)	< .001	0.2 (0.1;0.3)	< .001
46–60 years	0.3 (0.3;0.4)	< .001	0.3 (0.3;0.4)	< .001
61–75 years	0.5 (0.3;0.6)	< .001	0.4 (0.3;0.6)	< .001
76 years and over	0.5 (0.3;0.6)	< .001	0.5 (0.3;0.6)	< .001
Occupation		.017		.014
No, incapacitated to work	0.2 (0.1;0.2)	.001	0.2 (0.1;0.3)	.001
No, prepension	0.0 (−0.2;0.1)	.629	-0.1 (−0.3;0.1)	.336
No, pension	0.0 (−0.1;0.1)	.680	0.1 (−0.1;0.2)	.397
No, unemployed	0.0 (−0.1;0.1)	.726	0.0 (−0.1;0.2)	.692
No, student	0.0 (−0.1;0.1)	.372	0.0 (−0.1;0.1)	.522
No, homemaker	0.1 (0.0;0.2)	.184	0.1 (−0.1;0.2)	.276
No, never or not yet worked	−0.2 (− 0.5;0.1)	.113	− 0.3 (− 0.6;0.1)	.117
Yes	Ref	Ref	Ref	Ref
Confirmed infection with symptoms				
No	0.2 (0.1;0.3)	.001	0.2 (0.1;0.4)	< .001
Yes	Ref	Ref	Ref	Ref
Survey				
September 2020	0.1 (0.1;0.2)	< .001	0.1 (0.1;0.2)	< .001
April/May 2021	Ref	Ref	Ref	Ref

*Past adherence model: R*^*2*^ *= .076; adjusted R*^*2*^ *= .075*

*Future adherence model: R*^*2*^ *= .066; adjusted R*^*2*^ *= .062*

^a^Other characteristics that were also included as potential predictors, but did not have a significant association in either model, were annual income, score for health today, household composition and educational level.

^b^The sample size of both multivariate analyses was 3989.

## Discussion

To our knowledge, our study was the first to assess the perceptions of and adherence to COVID-19 measures in Belgium at multiple times, on a large total sample of nearly 4000 respondents representative of the adult population in terms of gender, age, region and socio-economic status,. The results of the surveys revealed that both reported understanding of the preventive measures and their perceived usefulness were higher at the second survey in April/May 2021 than at the first one in September 2020. This was particularly the case for measures that were implemented at both survey periods, namely wearing a face mask in public spaces and shopping with maximum one other person. At the time of the second survey, these measures had been in place for a long time, which may explain the fact that they were better understood and that citizens were more likely to consider them as useful. However, it is important to note that most measures differed between the two periods. The better understanding and perceived usefulness of the preventive measures at the time of the second survey could therefore also be due to other factors, such as a clearer and less ambiguous formulation or overall better communication about the reasons for the measures.

In contrast, both past adherence to the measures and (intended) future adherence were lower at the second survey period, compared to the first. For the two recurring measures, the decrease of the score for future adherence was rather small, especially with regard to shopping with maximum one other person. Since at the time of the second survey all measures had been in place for more than 6 months, it is likely that this caused a certain level of fatigue amongst citizens. This may especially apply to the measures involving a reduction of social contacts, which were the ones that received the lowest scores in the second survey. This is in line with reports from other Belgian studies, showing a lower motivation to adhere to COVID-19 measures in April and May 2021 than in September 2020 [27], and also indicating that people had more contacts outside their household in April/May 2021 than in September 2020 [28]. Being confined also had a negative impact on mental health of affected populations [29], particularly among women and younger age groups [7]. A study in the US showed a negative relationship between having mental health problems such as a social distance burn-out and depressive symptoms on the one hand, and adherence to COVID-19 measures on the other hand [30]. A survey from the National Institute of Health in Belgium showed high levels of anxiety and depression among the general population since the start of the COVID-19 pandemic, especially among people aged 18–29 [26]. Since in our study mental health was not assessed, we were not able to investigate the relationship between individual mental health and adherence.

Between the first and the second survey, there was a strong increase in the proportion of respondents that had experienced a confirmed COVID-19 infection. This is an expected finding, since, like most European countries, Belgium was confronted with an increasing number of cases between the two study periods [24]. Yet, while there was no difference in the perceived health consequences of COVID-19 for those who had had an infection, the expected health consequences reported by those who had not yet been infected at the time of the second survey was significantly higher than for the first survey. This may be related to the fact that at the second survey period, more people knew someone who had been infected: almost twice as many respondents knew someone who had been hospitalised with COVID-19, an important indicator of infection severity.

On the other hand, the respondents’ perceived risk of getting infected with COVID-19 was lower in the second survey than in the first, which may be explained by the fact that in April/May 2021 nearly a third (30%) of them had been vaccinated at least once. An unexpected finding however, is that the expected risk of older family members (parents and grandparents) being infected was higher in the second than in the first survey, especially since mainly older people had been vaccinated in Belgium at that time. Possibly, the fact that a larger proportion of respondents knew someone close who had been infected, sometimes with severe illness, might have made them more concerned about their own (vulnerable) relatives. However, this cannot be substantiated on the data from this study.

A difference was also observed in the support for the COVID-19 measures between the two study periods. The lower percentage of respondents who agreed with the statement that ‘the government should recommend, but not oblige the COVID-19 measures’ and the higher agreement with the statement that ‘the government should control the COVID-19 measures’ in the second survey suggests that citizens find it increasingly important to have clarity on what is expected from them, and that it should not be left up to the individual to decide this. Since COVID-19 had been part of people’s lives for more than a year in April/May 2021, less importance was given to reminders or ‘nudges’ for preventive action compared to September 2020. Arguably, this may be because these actions became habits that were integrated in everyday life, so that nudges became less necessary.

Our study identified several characteristics associated with lower levels of adherence in both surveys. The finding that men adhere less than women, and younger age groups less than older ones, are similar to those of studies in other countries that studied characteristics of lower adherence [7, 8, 16, 17]. Yet while previous research in Belgium also identified disadvantaged or lower socio-economic background as a risk factor for low adherence [8, 19], educational level and annual income were not found to be significant contributors for past or (intended) future adherence in our study. In terms of occupational status, the only group that differed significantly from the reference group of workers were those who were incapacitated, and their adherence levels were actually higher. Since those who are incapacitated to work have likely underlying health problems, they might feel more vulnerable to becoming infected with COVID-19, and as such adhere stricter to the measures in order to protect themselves. On the other hand, French-speaking citizens were less adherent and intent on future adherence than Dutch speakers, and inhabitants of Wallonia less than inhabitants of Flanders or the Brussels Capital region. These findings are highly correlated, as Wallonia is a French-speaking region of Belgium, Flanders is Dutch-speaking, and Brussels is both French- and Dutch-speaking. The reasons for these findings are not clear, but since almost 40% of Belgians have French as their native language [31], this important difference warrants further investigation. It does suggest, however, that adherence to measures against COVID-19 does not only depend on what is being decided on a national level, but that cultural and linguistic differences within the population have an impact as well.

The last group that had lower adherence levels consisted of those with a symptomatic, confirmed COVID-19 infection. We see three potential explanations for this: either this group feels protected against COVID-19 due to their previous infection, and therefore feels that they do not have to adhere to the rules; or this group consists of individuals that are less likely to adhere (because of lack of motivation or faced with environmental barriers that make measures more difficult to adhere to), and are therefore more prone to an infection; or this group has perceived milder symptoms, and the perceived severity of a COVID-19 infection is therefore lower for them. A qualitative study among those who have been previously infected could potentially provide more insight into this.

Of all the measures that were investigated in the second survey, the two measures related to social contact (‘having one close contact’ and ‘limiting close contact to one per household’) were seen as the most difficult to adhere to, both in the past and in terms of (intended) future adherence. These two measures are arguably the ones that are most restrictive for people’s daily lives. Since these measures had already been in force for over 6 months at the time of the second survey, the difficulty to adhere to them is not surprising. This is also in line with the result of a multi-country study that showed potential pandemic fatigue, and as a result lower adherence, over time for high-cost measures such as social distancing [23]. In contrast, a measure that received overall high scores in terms of understanding, perceived usefulness, ease to adhere and past and future adherence is the use of a face mask in public spaces. In fact, the scores for this measure even became more positive compared to the first survey, implying that this measure has been well implemented in Belgian society. The same is also observed for testing and quarantining for those who have symptoms, indicating the perceived importance of this measure by citizens.

The second survey also allowed to investigate the perceptions regarding vaccination. High scores were given in support of the statements that ‘COVID-19 vaccines are important to protect yourself and others’ and ‘it is important that everyone is vaccinated against COVID-19’, indicating that most people accept vaccines as an important protective measure. Nonetheless, scores for the statements ‘COVID-19 vaccines are safe’ and ‘COVID-19 vaccines are effective in preventing infection’ were much lower. Since the perceived safety of vaccines has been identified as an important predictor of vaccination intention [32], effective risk communication on vaccine safety is a crucial issue to improve actual uptake.

Our study had some limitations. First, while the samples from both surveys matched the predefined targets well in terms of gender, age, region and socio-economic group, there was a slight underrepresentation of respondents from the lowest socio-economic group. Obtaining an equal number of respondents from this group is often problematic, as they are less likely to participate in surveys. Secondly, citizens of Belgium who do not speak French or Dutch could not participate, since the survey questionnaire was only available in those two languages. However, this represents not more than 5% of the country’s population [31]. Thirdly, due to the anonymity of our questionnaire, we could not ascertain whether certain individuals participated in both surveys, which would have required a correction in the analytical approach. However, due to the methodology used by the market research and opinion poll company, this probably concerns only a marginal number of respondents, if any. Fourthly, although we obtained information on vaccination status, we could not include this as a potential predictor for adherence in the multivariate models. This is partly due to the fact that vaccination status was only relevant during the second survey (vaccines were not administered yet in Belgium during September 2020), and partly because only selected populations had been invited to get vaccinated at the time of the second survey (mainly elderly, healthcare professionals and chronically ill). As such, it is unlikely that vaccination status measured at that time would serve as a predictor for adherence. It is possible, however, that it would become a factor at a later stage, after everyone older than twelve years has received an invitation to get vaccinated.

## Conclusions

This article presents the results of a survey study regarding the perception of and adherence to COVID-19 measures, focussing on the comparison between two surveys (September 2020 and April/May 2021). While reported understanding and perceived usefulness of the measures were higher at the second survey, both past and (intended) future adherence to the measures decreased. Although the exact reasons for this reduction are not clear, the measures for which the reduction in adherence was the most significant were the ones that have a restrictive impact on social contacts, thus going against basic psychological needs of people. It is also important to consider the potential effect of fatigue among the population in relation to measures that are sustained for a long period of time. This may have bearings on the risk communication strategies that are used by authorities at different levels of government: whereas the focus is often on explaining the content of the measures, it is important to also put emphasis on communicating to the public *why* specific measures may help in reducing transmission, and *how* the measures may be applied in different contexts. As the measures involving, amongst others, social life restrictions, teleworking and use of a face mask were very similar to those implemented in other countries, we feel the results of this study can be informative for a broad, international audience.

## Data Availability

The dataset used during the current study are available from the corresponding author on request.

## Appendix 1

**Table 8:** Demographic characteristics of the study population in each of the two surveys

Characteristic	First survey		Second survey		Difference
	N	%	N	%	***p***-value
Gender					.780
Male	983	49.0	962	48.5	
Female	1024	51.0	1020	51.4	
Other	1	0.0	1	0.1	
Age					.361
18–30 years	407	20.3	396	20.0	
31–45 years	472	23.5	480	24.2	
46–60 years	522	26.0	494	24.9	
61–75 years	557	27.7	543	27.4	
76 years and over	50	2.5	70	3.5	
Province					.995
Antwerp	320	15.9	326	16.4	
Flemish Brabant	184	9.2	197	9.9	
Limburg	154	7.7	157	7.9	
West Flanders	219	10.9	216	10.9	
East Flanders	263	13.1	264	13.3	
Brussels Capital Region	206	10.3	194	9.8	
Walloon Brabant	74	3.7	70	3.5	
Hainaut	250	12.5	231	11.6	
Liège	206	10.3	193	9.7	
Luxembourg	50	2.5	49	2.5	
Namur	82	4.1	86	4.3	
Household composition					.053
Alone without children	474	23.6	407	20.5	
Alone with children	135	6.7	129	6.5	
Couple without children	655	32.6	736	37.1	
Couple with children	494	24.6	477	24.1	
With parents	229	11.4	211	10.6	
Live together / share a flat (e.g. friends, acquaintances)	21	1.0	23	1.2	

## Appendix 2

**Table 9:** Socio-economic characteristics of the study population in each of the two surveys

	First survey		Second survey		Difference
Characteristic	N	%	N	%	***p***-value
Educational level					.398
Primary or without diploma	62	3.1	54	2.7	
Lower secondary	240	12.0	259	13.1	
Upper secondary	810	40.3	774	39.1	
Superior short type and bachelors	420	20.9	391	19.7	
Long/university level superior	471	23.5	503	25.4	
Occupation					.224
Yes	920	45.8	888	44.8	
No, incapacitated to work	161	8.0	146	7.4	
No, pre-pension	33	1.6	34	1.7	
No, pension	530	26.4	557	28.1	
No, unemployed	80	4.0	91	4.6	
No, student	180	9.0	152	7.7	
No, homemaker	88	4.4	107	5.4	
No, never or not yet worked	16	0.8	8	0.4	
Net annual household income					.683
Less than EUR 15,000	164	8.2	148	7.5	
Between EUR 15,000 and 29,999	612	30.5	592	29.9	
Between EUR 30,000 and 44,999	534	26.6	553	27.9	
Moren than 45,000	319	15.9	333	16.8	
I do not know	379	18.9	357	18.0	

## Appendix 3

**Table 10:** Native language skills of the study population in each of the two surveys

	First survey		Second survey	
Language	N	%		
Dutch	1072	53.4	1062	53.6
French	793	39.5	796	40.1
English	29	1.4	10	0.5
Italian	25	1.2	14	0.7
Arabic	19	0.9	18	0.9
German	8	0.4	7	0.4
Sub-Saharan African language	4	0.2	5	0.3
Berber	3	0.1	2	0.1
